# Redox modulation of thimet oligopeptidase activity by hydrogen peroxide

**DOI:** 10.1002/2211-5463.12245

**Published:** 2017-06-19

**Authors:** Marcelo Y. Icimoto, Juliana C. Ferreira, César H. Yokomizo, Larissa V. Bim, Alyne Marem, Joyce M. Gilio, Vitor Oliveira, Iseli L. Nantes

**Affiliations:** ^1^ Departamento de BiofísicaUniversidade Federal de São PauloBrazil; ^2^ Laboratório de Nanoestruturas para Biologia e Materiais AvançadosCentro de Ciências Naturais e HumanasUniversidade Federal do ABCSanto AndréBrazil; ^3^ Present address: Structural Biology and Biophysical Chemistry LabNew York University Abu DhabiSaadiyat Marina District, Abu DhabiUnited Arab Emirates; ^4^ Present address: Departamento de NeurologiaCentro de DegeneraçãoUniversidade de São Paulo ‐ Escola de MedicinaSão PauloSPBrazil

**Keywords:** EC 3.4.24.15, hydrogen peroxide, protein oxidation, thioredoxin

## Abstract

Thimet oligopeptidase (EC 3.4.24.15, TOP) is a cytosolic mammalian zinc protease that can process a diversity of bioactive peptides. TOP has been pointed out as one of the main postproteasomal enzymes that process peptide antigens in the MHC class I presentation route. In the present study, we describe a fine regulation of TOP activity by hydrogen peroxide (H_2_O_2_). Cells from a human embryonic kidney cell line (HEK293) underwent an ischemia/reoxygenation‐like condition known to increase H_2_O_2_ production. Immediately after reoxygenation, HEK293 cells exhibited a 32% increase in TOP activity, but no TOP activity was observed 2 h after reoxygenation. In another model, recombinant rat TOP (rTOP) was challenged by H_2_O_2_ produced by rat liver mitoplasts (RLMt) alone, and in combination with antimycin A, succinate, and antimycin A plus succinate. In these conditions, rTOP activity increased 17, 30, 32 and 38%, respectively. Determination of H_2_O_2_ concentration generated in reoxygenated cells and mitoplasts suggested a possible modulation of rTOP activity dependent on the concentration of H_2_O_2_. The measure of pure rTOP activity as a function of H_2_O_2_ concentration corroborated this hypothesis. The data fitted to an asymmetrical bell‐shaped curve in which the optimal activating H_2_O_2_ concentration was 1.2 nM, and the maximal inhibition (75% about the control) was 1 μm. Contrary to the oxidation produced by aging associated with enzyme oligomerization and inhibition, H_2_O_2_ oxidation produced sulfenic acid and maintained rTOP in the monomeric form. Consistent with the involvement of rTOP in a signaling redox cascade, the H_2_O_2_‐oxidized rTOP reacted with dimeric thioredoxin‐1 (TRx‐1) and remained covalently bound to one subunit of TRx‐1.

**Abbreviations**
Abz: o‐aminobenzoyl
EDDnp: N‐[2,4‐dinitrophenyl]‐ethylenediamine
JA‐2: N‐[1‐(R,S)‐carboxy‐3‐phenylpropyl]Ala‐Aib‐Tyr‐p‐aminobenzoate
RLMt: rat liver mitoplasts
TOP: thimet oligopeptidase
TRx‐1: thioredoxin‐1

Thimet oligopeptidase (TOP, 1 3.4.24.15) is a 77‐kDa zinc metalloendopeptidase that presents the HE*XX*H motif in the active site 1, 2, 3, 4. TOP is present in a diversity of mammalian tissues. The highest levels of TOP expression have been reported to occur in the brain, pituitary gland, and testis 2, 5, 6. In cells, according to the cell type, TOP has been identified in the cytosol, membranes, and nucleus, which is consistent with a diversity of physiological roles 2, 7. TOP hydrolyzes many bioactive peptides of the central nervous system and other tissues 6 including bradykinin (BK), neurotensin (NT), opioid peptides, angiotensin I and gonadotrophin‐releasing hormone 8, 9, 10, 11. TOP also cleaves several postproteasomal peptides 12, resulting from the proteolytic activity against endogenous oxidized proteins (self‐antigens) as well as on foreign antigens (from bacteria and viruses). Therefore, TOP inhibition favors the cell‐surface presentation of MHC‐I (major histocompatibility complex‐I), in a mechanism that has been described to be dependent on ROS (reactive oxygen species) production 13, 14, 15. TOP has also been demonstrated to handle processing of the melanoma antigen PRAME peptide in a proteasome‐independent mechanism 16. Recently, TOP expression was positively correlated with tumor malignancy both in hepatocellular carcinoma and lung cancer, making its expression a good predictive prognostic factor for the tumor evolution 17, 18. TOP activity is related to increased degradation of the Aβ peptide, the component of amyloid plaques in Alzheimer's disease produced by the degradation of the APP (amyloid precursor protein) 8, 19. The regulation of TOP activity occurs at the level of expression and by posttranslational modification such as phosphorylation 20, 21. Another mechanism postulated as contributing to TOP regulation is a multimerization by the formation of intermolecular disulfide bonds. TOP multimerization was described to occur *in vitro,* in the absence of reductant 3, 22, and also in cellular assays due to the glutathionylation of the Cys246 and Cys175 23, 24. In fact, a distinguishing feature of this peptidase is its activation by reducing agents, from which the denomination thimet (thiol‐metallo) derives. TOP contains 15 cysteine residues that do not form intramolecular disulfide bonds. Of these 15 residues, six are surface exposed Cys that can form interchain disulfide bonds. Among the six surface exposed Cys residues, the cluster of cysteines 246, 248 and 253 (Cys246/248/253) are the most important residues for TOP oligomerization–inactivation 3. We identified the modulation of the TOP activity in HEK293 cells by changes in H_2_O_2_ concentrations after exposure to a hypoxia/reoxygenation treatment. Considering the well‐known signaling role of H_2_O_2_ we investigate the redox modulation of TOP activity by H_2_O_2_. Beyond this TOP oligomerization–inactivation process, we could demonstrate that H_2_O_2_ can reversibly oxidize TOP thiols to sulfenic and sulfonic acids, which in turn can be recycled by regulatory reductases, modulating the activity of monomeric TOP.

## Results

### TOP activity in HEK293 cells increases immediately after 12‐h hypoxia and decreases after 2‐h reoxygenation

**Figure 1:**
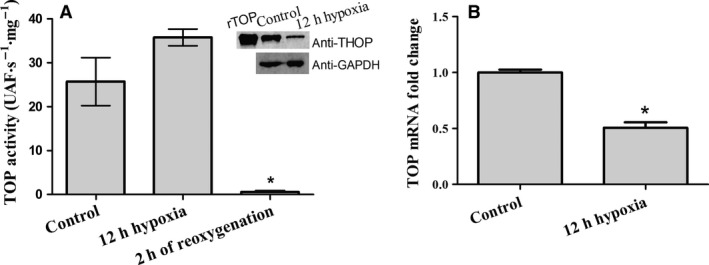
HEK293 cells submitted to hypoxia (12 h) or hypoxia (12 h)/reoxygenation (2 h) conditioning. (A) TOP‐like specific activity measured in HEK293 cell extracts. TOP‐like activity is the hydrolysis velocity of the FRET substrate Abz‐GFSPFRQ‐EDDnp inhibited by the specific inhibitor, JA‐2. After hypoxia TOP‐like activity measured presented a slight increase however after 2 h of reoxygenation, TOP specific activity strongly decreased (*P < 0.01). Inset of A. Western blot using anti‐TOP specific antibody. After hypoxia the TOP protein detected decreased about 2.5‐fold as indicated by the western blot densitometry. (B) SYBR Green qPCR using specific primers for TOP mRNA. 2^−ΔΔ^ ^CT^ analysis indicates a decrease of 50% in TOP mRNA. Control cells exhibited any significant difference between the mean value of TOP activity measured after 12 and 14 h of incubation as well as any difference in the TOP mRNA measured by the qPCR and also in the observed protein detected by the anti‐TOP in the western blot assays. Data are shown as mean ± standard deviation (*n* = 3) *P < 0.01.

Human embryonic kidney cells (HEK293 cell line) were submitted to an ischemia/reperfusion conditioning that leads to H_2_O_2_ production. TOP specific activity was determined using the substrate Abz‐GFSPFRQ‐EDDnp and the inhibitor JA‐2 in extracts of treated (12‐h hypoxia or 12‐h hypoxia followed by 2‐h normoxia/reoxygenation) and control cells (12‐h or 14‐h normoxia). It is important to note that to access the cells just after hypoxia an immediate reoxygenation also occurs, because the transfer from the hypoxia chamber to the regular incubator (normoxia condition), where the cells were immediately processed. Figure 1A shows that after hypoxia HEK293 cells exhibited a mean of 32% increase of TOP activity, despite the fact that on opposite way, both TOP mRNA detected by RT‐qPCR (Fig. 1B) as well as the TOP protein level detected with anti‐TOP antibody in western blot analysis (inset of Fig. 1A) decreased. TOP activity determined for samples of HEK293 cells after 2 h of reoxygenation revealed a mean of 90% decrease of the enzyme activity. The TOP activity decrease detected in HEK293 cells after 2‐h reoxygenation did not have the contribution enzyme expression as no difference was detected between the samples 12‐h hypoxia and 12‐h hypoxia/2‐h reoxygenation by RT‐qPCR as well by western blots with anti‐TOP antibody (data not shown). Also, HEK293 cells submitted to hypoxia followed by 2‐h reoxygenation produced 1 μm of H_2_O_2_ measured by Amplex Red assay. This result suggests the association of TOP activity modulation in HEK293 cells with the H_2_O_2_ production. Furthermore, considering that other factors related to hypoxia and reoxygenation might respond for the TOP modulation, we first pursued in the analysis of the direct effect of H_2_O_2_ on TOP activity, using isolated rat liver mitoplasts (RLMt) and a solution of purified rat TOP (rTOP).

### H_2_O_2_ generated by poisoned isolated rat liver mitoplasts increases rTOP activity

**Figure 2:**
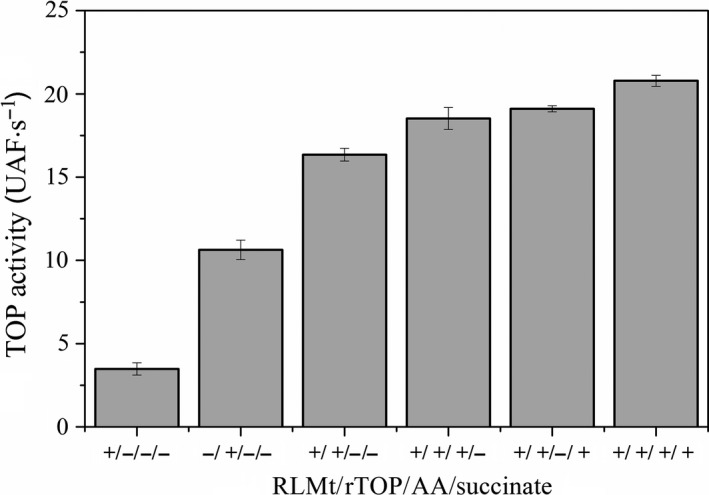
Effect of RLMt (rat liver mitoplasts) bioenergetics on rTOP (recombinant thimet oligopeptidase) activity. rTOP activity is the decrease of the rate of FRET substrate Abz‐GFSPFRQ‐EDDnp hydrolysis promoted by the specific inhibitor, JA‐2. Pure RLMt extracts showed TOP‐like activity. Antimycin A (AA) and succinate stimulate the H_2_O_2_ production and increase rTOP activity.

The *ex vivo* approach consisted of the addition of rTOP to a suspension of rat liver mitoplasts in the absence and the presence of antimycin A, succinate, or antimycin A plus succinate. Antimycin A promotes inhibition of the complex III of the respiratory chain leading to electron escape from complex III and formation of superoxide ion. The addition of succinate, which contributes to maintaining complex III reduced, exacerbated the formation of superoxide ion. Superoxide ion produced by RLMt (rat liver mitoplasts) further disproportionated to H_2_O_2_ by the action of manganese superoxide dismutase 25. Figure 2 shows a basal proteolytic activity of RLMt suspension (1 mg protein·mL^−1^) that might be assigned to mitochondrial neurolysin. The activity of rTOP solution increased more than 50% in the presence 1 mg protein·mL^−1^ RLMt suspension. The increase of rTOP activity was not a sum of independent RLMt and rTOP activities. It is not possible to estimate the contribution of neurolysin activity in this condition. However, it is expected that a predominant contribution of rTOP activity in the system liver RLMt/rTOP due to competition with neurolysin activity. Furthermore, even subtracting the basal contribution of the independent RLMt proteolytic activity, an increase of ~20% rTOP activity in the presence of RLMt persisted. The addition of antimycin A or succinate, both responsible for an increase in electron escape from the respiratory chain, led to a ~80% increase in the rTOP activity. The increase of rTOP activity in the presence of antimycin A or succinate should be ~50% by subtracting the basal contribution of RLMt. In the simultaneous presence of succinate and antimycin A, an increase of ~100% in rTOP activity was observed that should be estimated around 80% increased by subtracting RLMt basal activity.

Considering the results obtained in cells (*in vivo*) and with RLMt (*ex vivo*), it was investigated the direct effect of different concentrations of H_2_O_2_ on rTOP activity.

### H_2_O_2_ concentration can modulate rTOP activity

**Figure 3:**
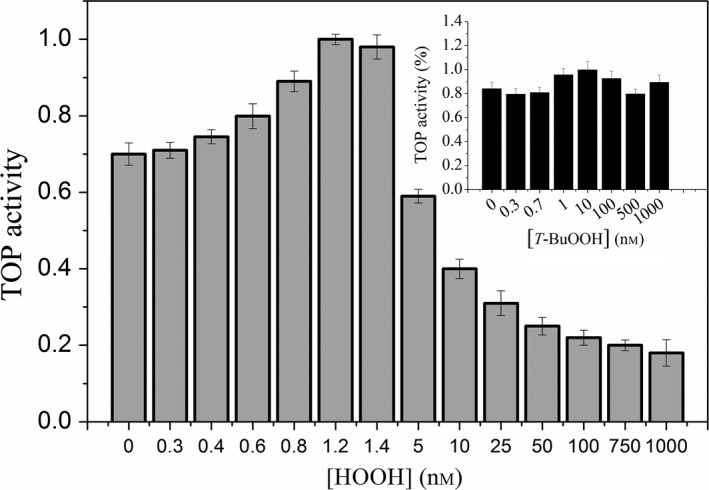
Relative recombinant rTOP activity determined in the absence or different H_2_O_2_ concentrations. Inset: Relative recombinant rTOP activity determined in the absence or different *t*‐butyl hydroperoxide (*T*‐BuOOH) concentrations. rTOP demonstrated to be activated by H_2_O_2_ and to be insensitive to *T*‐BuOOH.

Hydrolysis of the substrate Abz‐GFSPFRQ‐EDDnp by 0.1 nmol·L^−1^ of rTOP determined in the absence and the presence of different H_2_O_2_ concentrations are shown in Fig. 3. The maximal activation, an increase of 42% about the control activity appeared at 1.2 nmol·L^−1^. A progressive inhibition of the activity (15 to 75%, respectively) appeared at the concentration range of 5 to 1000 nmol·L^−1^ of H_2_O_2_ added to the medium (Fig. 3). The curve of the modulation of rTOP activity as a function of H_2_O_2_ concentration *in vitro* reinforced that endogenously produced H_2_O_2_ is responsible for the rTOP activation observed in the *in vivo* and *ex vivo* assays. The peroxide modulation of rTOP activity is specific for H_2_O_2_ as *t*‐butyl hydroperoxide (*t*‐BuOOH) did not affect significantly rTOP activity (inset Fig. 3).

### H_2_O_2_ oxidizes TOP leading to higher oxidative states of cysteine residues

**Figure 4:**
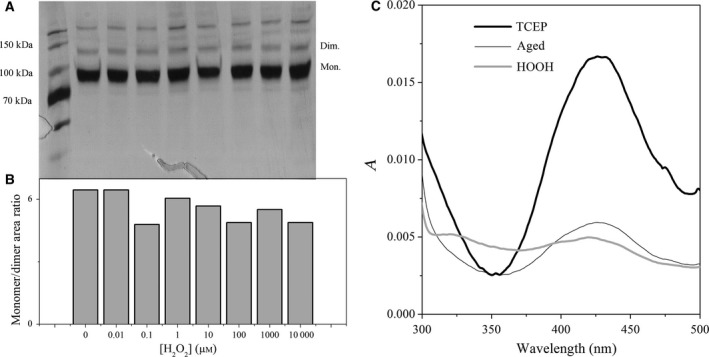
Pure H_2_O_2_‐promoted recombinant rTOP oxidation without interfering in its oligomeric state. (A) SDS/PAGE of rTOP treated, from the left to the right with [H_2_O_2_] concentrations of 0, 0.01, 0.1, 1, 10, 100, 1000 and 10 000 μm. (B) TOP monomer/dimer + trimer ratios determined by densitometry of SDS/PAGE that is shown in A. (C) Electronic absorption spectra of rTOP submitted to different treatments followed by reaction with NBD‐Cl. The thin solid line corresponds to the spectrum of aged rTOP; thick solid line corresponds to the spectrum of TCEP reduced rTOP and the gray line to the spectrum of rTOP treated with H_2_O_2_.

Considering that H_2_O_2_ modulates TOP activity in a concentration‐dependent manner, a question arose: what kind of oxidative changes cause TOP activation and inhibition? In the native TOP structure, the internal cysteine residues do not form intramolecular disulfide bonds and in aged and air‐oxidized TOP the external cysteine residues establish interchain disulfide bonds leading to TOP oligomerization and inhibition 3, 22. In the case of H_2_O_2_‐oxidized rTOP, the SDS/PAGE demonstrated that even in inhibitory concentrations, H_2_O_2_ did not promote rTOP oligomerization (Fig. 4A,B). Figure 4A shows that the bands of monomeric, dimeric and trimeric rTOP did not present significative changes after the treatment with different H_2_O_2_ concentrations. The incapacity of H_2_O_2_ to promote rTOP oligomerization was corroborated by the data of monomer/oligomer ratio obtained by densitometry (Fig. 4B). Therefore, the redox state of cysteine residues was investigated using the reagent NBD‐Cl (7‐nitrobenz‐2‐oxa‐1,3‐diazole chloride) that has a specific absorbance spectrum peaking at 347 nm when linked to sulfenic acid. Figure 4C, thin solid line, shows that the reaction of air‐oxidized native rTOP with NBD‐Cl yielded a sample with an absorbance spectrum peaking at 420 nm assigned to rTOP‐S‐NBD. This spectrum did not exhibit bands in the 300‐ to 350‐nm region. When rTOP aged and oxidized in the air was reduced by the reagent TCEP (tris(2‐carboxyethyl)phosphine), a significant increase of the band peaking at 420 nm was observed (Fig. 4C, thick solid line). That is consistent with the conversion of cysteine residues involved in the inter‐chain disulfide bonds to reduced cysteine with –SH groups that are available for the formation of rTOP‐S‐NBD adducts. The sample of rTOP treated with 100 μm H_2_O_2_ exhibited a decrease in the absorbance at 420 nm concomitant with the increase of absorbance at 320–350 nm (Fig. 4C, gray line). These results are consistent with the formation of a rTOP‐SO‐NBD adduct.

**Figure 5:**
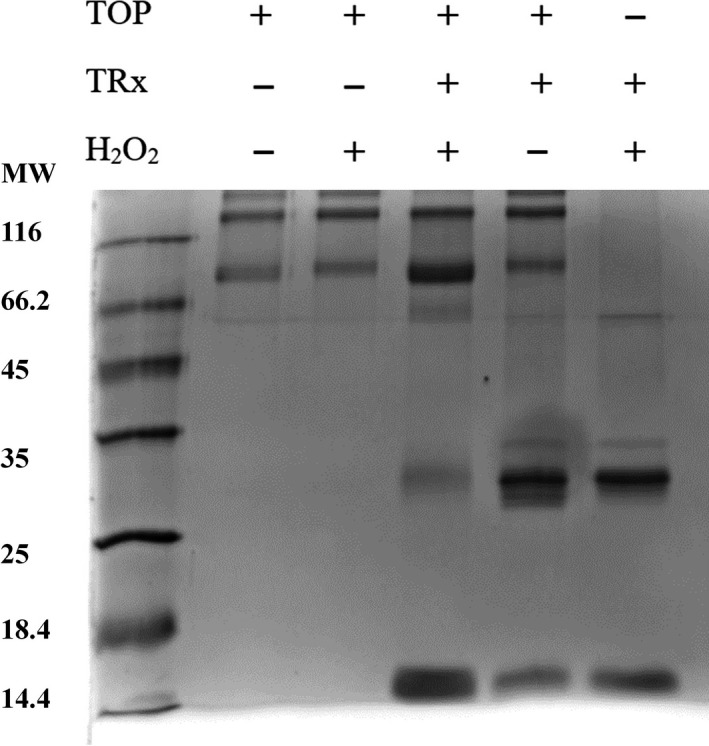
SDS/PAGE (nondenaturant conditions) of TRx reacted with recombinant rTOP in the native form and after treatment with H_2_O_2_ as indicated in the Figure.

**Figure 6:**
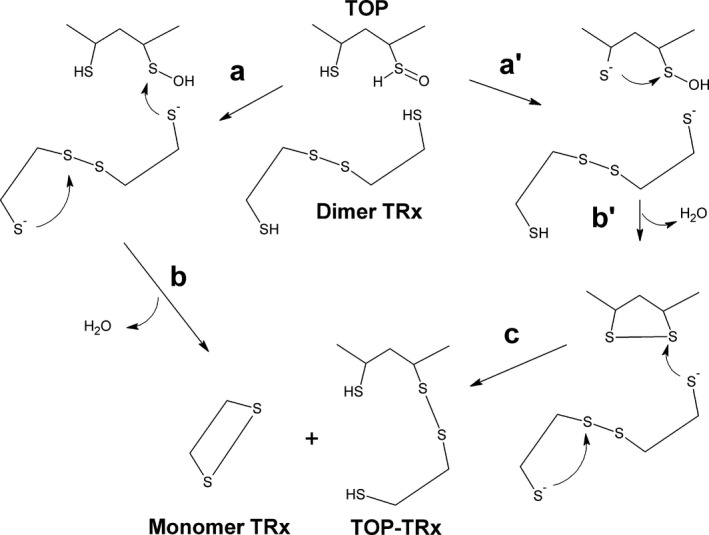
Schematic representation of the reaction routes between TOP‐SOH and TRx leading to covalent complex TOP‐TRx. Step *a*—Nucleophilic attack of the rTOP sulfenic acid by a thiolate of a subunit of dimeric TRx; step *b*—the structural change of TRx promoted by the ligation of TOP leading to an interchain/intrachain disulfide exchange and formation of a rTOP‐TRx covalent complex and the liberation of a TRx monomer. Steps *a′* and *b′*—a cysteine residue of rTOP acting as a resolving cysteine leading to the formation of a disulfide bond. Step *c*—the rTOP disulfide bond should be a target for the nucleophilic attack of a TRx1 thiolate followed by the occurrence of an interchain/intrachain disulfide exchange.

The modulation of rTOP activity by H_2_O_2_, a signaling molecule suggested that rTOP could participate in a signaling redox cycle. The involvement of rTOP in a redox cycle requires that enzymes such as thioredoxin could reverse the oxidative changes. Further, the formation of sulfenic acid, a reversibly oxidized thiol derivative 26, in conditions of rTOP activation by H_2_O_2_ reinforced a possible participation of rTOP in a redox cycling. Therefore, native rTOP and H_2_O_2_‐treated rTOP were incubated with thioredoxin (Fig. 5). For a control condition, thioredoxin (TRx) was also incubated with H_2_O_2_. In the following the samples were submitted to SDS/PAGE. In the lane corresponding to the running of H_2_O_2_‐oxidized rTOP and incubated in the presence of TRx, a significant increase of the band containing the monomeric TRx was observed in detriment of the band containing the dimeric TRx. In this lane, a higher intensity of the band corresponding to monomeric TOP was observed without changes in the intensity of the band corresponding to its dimeric form. Native rTOP and H_2_O_2_ did not promote significant changes in the intensity of TRx bands. The results obtained by SDS/PAGE suggest two possible mechanisms, depicted in Fig. 6. According to Fig. 6 in a putative noncovalent complex of dimeric TRx and rTOP‐SOH, the first step could involve nucleophilic attack of the rTOP sulfenic acid by a thiolate of a subunit of dimeric TRx (step *a*). The structural change of TRx promoted by the ligation of TOP could lead to a reaction of interchain/intrachain disulfide exchange (step *b*) with consequent formation of a rTOP‐TRx covalent complex and the liberation of a TRx monomer. Another possibility is that in the putative noncovalent complex of dimeric TRx and rTOP‐SOH, a cysteine residue of rTOP can act as a resolving cysteine leading to the formation of a disulfide bond (step *a′* and *b′*). The rTOP disulfide bond should be a target for the nucleophilic attack of a TRx1 thiolate followed by the occurrence of an interchain/intrachain disulfide exchange (step *c*). In cells, the formation of TRx‐rTOP complex could be reduced by thioredoxin reductase (TRxR). The latter mechanism is similar to that described for the reduction of peroxiredoxin (Prx) by TRx1 27. TRx1 uses the free thiols of Cys32 and Cys35 to catalyze the selective reduction of disulfide bonds of target proteins. However, TRx1 can also reduce cysteine sulfenic acids probably using the same catalytic residues, that is Cys32 and Cys35 28. The possible conversion of dimeric TRx1 to the monomeric form raises the possibility of further redox regulation processes that deserves future investigations.

### Activity of TOP C246S, C248S, and C253S triple mutant is not affected by H_2_O_2_

**Figure 7:**
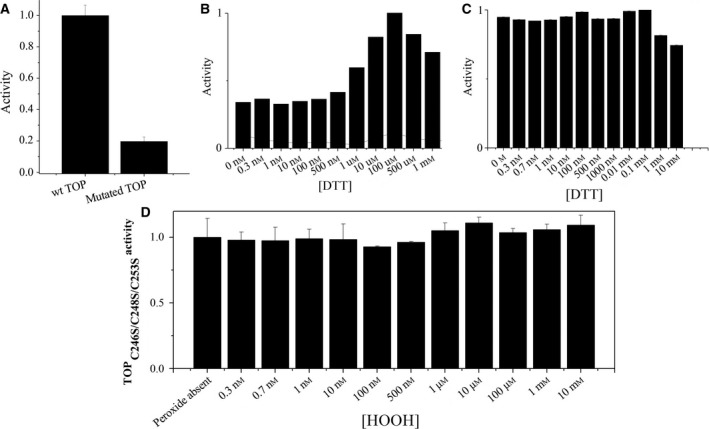
The C246S/C248S/C253S mutations abolish the DTT and H_2_O_2_ effects on rTOP activity. (A) Wild‐type rTOP activity compared with rTOP C246S/C248S/C253S triple mutant activity in the same conditions. (B) Wild‐type rTOP activity determined in the absence or the of different DTT concentrations. (C) rTOP C246S/C248S/C253S triple mutant activity determined in the absence or the of different DTT concentrations. (D) rTOP C246S/C248S/C253S triple mutant activity determined in the absence or in the presence of different H_2_O_2_ concentrations (compare D with Fig. 3).

The cysteine residues Cys246/248/253 are important in the rTOP oligomerization–inactivation process because these residues take part in intermolecular disulfide bonds 3. Therefore, we investigated the participation of these residues in the modulation of rTOP activity by H_2_O_2_. For this purpose, a rTOP mutant with these three cysteine residues replaced by serine residues was used. Figure 7A shows that triple‐mutated rTOP C246S, C248S, and C253S possesses only 20% of the activity of WT (wild‐type) rTOP. Unlike WT rTOP (Fig. 7B), the activity of the triple‐mutated rTOP is not affected by DTT (1,4‐dithiothreitol; Fig. 7C). Similarly, H_2_O_2_ had no effect on triple‐mutated rTOP (Fig. 7D). This result is consistent with the key residues Cys246, Cys248, and Cys253 as the target for the modulatory effect of H_2_O_2_ on rTOP activity. Taken together, the results suggested that modifications at the cluster formed by the Cys residues 246, 248, and 253 affect rTOP activity, even if this modification does not lead to rTOP dimerization/oligomerization. We discarded possible interferences of the H_2_O_2_ treatment in the activity assays used herein through H_2_O_2_ treated rTOP kinetic assays with the FRET substrate Abz‐GFSPFRQ‐EDDnp and, inhibitory assays with the JA‐2 inhibitor (data not shown). The structural rearrangements of TOP that leads to allosteric regulation remain unexplained because these residues are quite distant from the enzyme active site. Nevertheless, in fact, the structural basis to explain the inactivation of dimeric TOP‐TOP leads to the same question.

## Discussion

**Figure 8:**
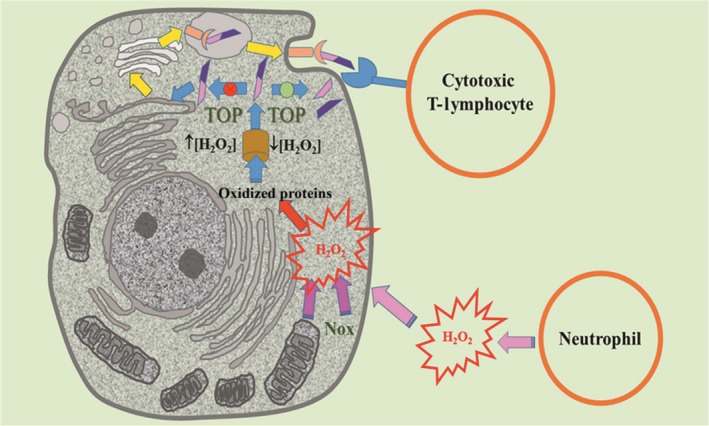
Regulation of TOP activity by H_2_O_2_ concentration in cells.

Hydrogen peroxide (H_2_O_2_) is a long‐lived and nonradical pro‐oxidant molecule initially related to oxidative damages and now also related to cell signaling by a substantial body of evidence. The recent studies demonstrating that aquaporin‐3 regulates the transmembrane traffic of H_2_O_2_ in addition to the specificity for signaling targets refuted the previous common idea of H_2_O_2_ as a freely diffusible molecule in cells and tissues. In fact, a diversity of recent studies has described H_2_O_2_ stabilized in cell compartments 29. A diversity of mechanisms produces H_2_O_2_ in different cell compartments. Electron escape from respiratory complexes in mitochondria was the early source described for H_2_O_2_ generation in cells. Xanthine oxidase, lipoxygenase, and myeloperoxidase are also important sources of the generation H_2_O_2_ 30, 31. More recently the enzymes of the Nox (NADPH oxidase) family emerged as a source of H_2_O_2_ strongly related to signaling mechanisms. The Nox family enzymes firstly described as associated with the plasma membrane of phagocytic cells are now identified in a diversity of subcellular compartments of nonphagocytic cells such as mitochondria, nucleus, and endoplasmatic reticulum. Furthermore, a single cell type can express different Nox isoforms targeted to several subcellular compartments 32, 33. Literature data have shown that cysteine residues of different proteins are likely the principal target of the signaling action of H_2_O_2_. The redox signaling on cysteine residues requires reversible oxidation of thiol group to sulfenic acid intermediates (Cys‐SOH) 34. The irreversible oxidation of cysteine residues to sulfinic and sulfonic acid has been associated with injury and cell death 35, 36. The protein tyrosine phosphatases (PTP) are well‐known targets of H_2_O_2_ generated by Nox 37. Another family of enzymes regulated by ROS is the family of matrix metalloproteinases (MMPs) 38. These enzymes have their expression and activation regulated by ROS. Interestingly, in the present study, we observed a fine regulation of a cellular metalloproteinase by H_2_O_2_. TOP plays an important role in the presentation of MHC‐I by cells. The degradation of irreversibly oxidized proteins by proteasomes generates a diversity of peptides that are almost all digested by cytosolic peptidases (Fig. 8). The peptides that escape from the degradation in the cytosol are translocated by the TAP (transporter associated with antigen processing) complex from the cytosol into the endoplasmic reticulum (ER). In the ER, the antigenic peptides bind the major histocompatibility complex class I (MHC class I) and are transported to the cell surface to be recognized by antibodies on the surface of lymphocyte T (Fig. 8). Unlike the cell proteins responsible for the production of antigenic peptides, TOP is not regulated by interferon‐γ. Therefore, considering the probable significant participation of TOP in the presentation of MHC class I at the cell surface and other signaling functions not yet elucidated it is reasonable to infer that this enzyme has a regulation mechanism. The synthesis of TOP occurs in cytosolic ribosomes without pre or pro inactive forms and further, no endogenous TOP inhibitors have been identified. Therefore, the regulation of TOP has been searched for at the transcriptional level 20, 21. On the other hand, considering the high thiol content of TOP, it is also reasonable to infer a regulation for this enzyme by a thiol‐centered redox mechanism. The results presented here showing a short‐term activation and long‐term inhibition of TOP in cells submitted to a hypoxia/reoxygenation model (*in vivo* assay), an activation of TOP in the presence of RLMt stimulated to increase H_2_O_2_ production (*ex vivo* assay) and TOP activation by nanomolar concentrations of H_2_O_2_ support H_2_O_2_ as the key molecule for the control of TOP activity control. Therefore, the present study brings to light the following questions about TOP: (a) What is the role of the high content of cysteine residues as they do not participate in the catalytic process? (b) How can a cytosolic enzyme acting in a reducing microenvironment be regulated by dimerization stabilized by disulfide bonds? (c) How does dimerization via disulfide bonds correlate with a probable control of TOP at the gene expression level? At least, Cys246, Cys248, and Cys253 seem to be involved in redox regulation of TOP. The present results by linking TOP regulation with a H_2_O_2_ concentration in cells suggest that the formation of sulfinic, sulfenic and sulfonic acids in addition to the interchain disulfide bonds are responsible for the control of the metalloproteinase activity. To identify the presence of higher forms of oxidation on TOP, we used the NBD trap, which maximally absorbs near 337 nm when attached to SOH as S‐O‐NBD. When H_2_O_2_ mildly oxidizes TOP_,_ we note an increase in this band. When we overoxidized rTOP, this absorption decreased, suggesting that higher oxidized forms were generated as NBD is unable to react with these modifications. When tested against Cys mutants both oxidative and reducing profiles were abrogated. The peculiar structure of TOP, bearing 15 cysteine residues some of them surrounded by acidic and basic residues, makes this enzyme a candidate to be a H_2_O_2_ sensor. TOP has the majority of its Cys residues located on the internal side of the structure but in an arrangement unfavorable to form internal disulfide bonds. However, higher oxidative states of cysteine may occur regarding the generation of sulfur–oxygen bonds. These states are the unstable (naturally reversible) sulfenic acid (SOH) that is formed by low levels of H_2_O_2_ in cells and the sulfinic (SO_2_) and sulfonic (SO_3_) acids, the two latter are associated with conditions of oxidative stress with high production of H_2_O_2_ and naturally irreversible. These cysteine derivatives are produced through the reduction of H_2_O_2_ by the low p*K*_*a*_ cysteine residues of key proteins. Subsequently, they are recycled to the SH form by the action of specific enzymes in events that composes the signaling pathway of H_2_O_2_. We discard the effect of TOP oligomerization in H_2_O_2_‐treated TOP by following SDS/PAGE of monomeric TOP. TOP oligomerization was reported in HEK293 challenged by 100–400 μm of H_2_O_2_ 23. H_2_O_2_ is diffusible across membranes, but studies of Choi *et al*. 39 demonstrated that exogenously added H_2_O_2_ has been shown to be less effective for triggering signaling responses than endogenously produced H_2_O_2_. In fact, the mechanisms of H_2_O_2_ deactivation present in cells suggest that the signals charged in this molecule are only transmitted over relatively short distances 40. The present results obtained *in vivo*,* ex vivo* and *in vitro* strongly raises the possibility that the action of this peptidase can be modulated by the redox state of cells. Higher oxidative forms of cysteine are becoming important physiologically and postulated as a chemical modification that links protein function to the oxidative status of cells. For example, the formation of a stabilized cysteine sulfinic acid in the DJ‐1 protein is critical for the mitochondrial function of this protein associated with inherited Parkinsonism 41. Thus, our results suggested that modifications at the cluster formed by the Cys residues C246, C248, or C253 affects TOP activity, even if this modification does not lead to TOP dimerization/oligomerization. The exact structural rearrangements that occur in the enzyme that explains this allosteric regulation remains to be elucidated. The decrease of TOP expression in response to the short‐term activation of the TOP cell content after reoxygenation links the chemical and structural modulation of TOP activity with the regulation of the activity of this enzyme at the level of gene expression. Figure 8 illustrates a possible mechanism to regulate MHC class I presentation via inhibition or activation of TOP according to the concentration of H_2_O_2_ in cells. H_2_O_2_ generated by an exogenous source such as neutrophils or by endogenous sources such as electron escape from respiratory chain and enzymes of the Nox family promotes direct or indirect oxidation of proteins. Proteasomes degrade the oxidized proteins generating peptides. These peptides could have a diversity of signaling functions including the participation in the MHC class I. In conditions in which the increase of H_2_O_2_ in the cell is relatively low, TOP is activated, promotes the proteolysis of the peptides and impairs the presentation in the MHC class I. Otherwise, when the H_2_O_2_ concentration is relatively higher, TOP is inhibited, and the peptides follow the secretory pathway. In the former condition, that is when the H_2_O_2_ concentration increases slightly in cells, TOP oxidation results in the formation of sulfenic acid and the enzyme activation could be reverted by TRx/TRxR (thioredoxin reductase) system. On the other hand, TOP irreversibly oxidized by high concentrations of H_2_O_2_ could be directed to proteolysis in the proteasome. It is important to note that H_2_O_2_ produced in cells submitted to hypoxia and reoxygenation could be produced by the electron escape from the respiratory chain as well as from Nox activity in different cell compartments. Hereafter, other studies must be conducted to elucidate the most significant source of H_2_O_2_ that can modulate TOP activity.

The relationship of the cysteine‐rich structure of TOP with its biological role and regulation has been a challenge, particularly the unfeasible regulation by enzyme oligomerization. Taken together, the results presented here demonstrate for the first time that TOP, a thiol‐rich enzyme, can potentially participate in cell signaling mediated by H_2_O_2_ with participation of thioredoxin. The conclusion is supported by the fine oxidative regulation of TOP promoted by H_2_O_2_‐induced formation of sulfenic acid that was demonstrated *in vivo*,* ex vivo,* and *in vitro* assays.

## Materials and methods

### Cell culture

HEK293 cell line was cultured in DMEM (Vitrocell, São Paulo, Brazil) containing 10 μg·mL^−1^ of streptomycin (Vitrocell) and supplemented with 10% fetal bovine serum (Vitrocell). HEK293_TOP cells which overexpress TOP protein were previously generated 42 and cultured under the same conditions.

### Hypoxia/reoxygenation assay

Cells were seeded in triplicate, at a concentration of 7 × 10^6^ cells (70% of confluence) in T‐75 bottles (Corning Incorporated, Corning, NY, USA), in 10 mL culture medium. The cultured cells were grown for 24 h before the experiments at 37 °C in a humidified 5% CO_2_ incubator. Subsequently, the cell culture medium was removed, and the cells were washed once with PBS. In the following, cells were incubated in two different conditions: 12 h in the normal atmosphere (control) and 12 h in a hypoxia chamber under 5% O_2_ atmosphere 43. A model of ischemia and reperfusion consisted in maintaining cultured for 12 h in a hypoxic chamber with subsequent return to the normal atmosphere. In this chamber, the atmosphere composition was 5% O_2_, 5% CO_2,_ and 90% N_2_, followed by a 2‐h incubation in a regular incubator with atmosphere composition of 30% O_2,_ 5% CO_2,_ and 65% N_2_. TOP activity was measured immediately after the removal of the hypoxia chamber condition and after 2 h of incubation under a normal atmosphere.

### Rat liver mitoplasts

Mitochondria were isolated by conventional differential centrifugation from the livers of adult Wistar rats weighting approximately 180 g and receiving food and water *ad libitum* in a light‐controlled room (12 h light/dark cycles). Each liver was reached through a bilateral abdominal incision. All procedures here were conducted according to an approved institutional animal experimentation protocol (committee of ethical research approval number 0540/11), which follows the ‘Guide for the Care and Use of Laboratory Animals in Research’ (in accordance with the National Institutes of Health, USA). The homogenate was prepared in 250 mm sucrose, 1.0 mm EGTA, and 5.0 mm HEPES buffer (pH 7.2). The mitochondrial suspension was washed twice in the same medium containing 0.1 mm EGTA, and the final pellet was diluted in 250 mm sucrose to a protein concentration of 80–100 mg·mL^−1^ 44, 45. RLMt (mitochondria devoid of the outer membrane) were prepared as described by Pedersen *et al*. 46, 47, 48. Rat liver mitoplasts were incubated in 50 mm Tris buffer, 100 mm NaCl, pH 7.4, at 37 °C. When indicated, the medium contained 2.5 mm succinate and 5 μm antimycin A. For every specified condition, rTOP was added to the RLMt‐containing medium and the TOP specific enzymatic activity determined using the fluorescent substrate Abz‐GFSPFRQ‐Eddnp (10 μm) 49 and the inhibitor JA‐2 (10 μm) 50, 51.

### Quantification of H_2_O_2_ produced by RLMt

Production of H_2_O_2_ was determined in 0.125 or 0.25 mg protein·mL^−1^ of mitochondrial suspensions in 10 mm KCl, 2 mm HEPES pH 7.4 52. The production of H_2_O_2_ was stimulated by the presence of 2.5 mm succinate, and 5 μm antimycin A. H_2_O_2_ was quantified by Amplex Red (Sigma‐Aldrich‐Merck, Darmstadt, Germany) in the concentration of 25 μm 53, 54. Briefly, Amplex Red is oxidized to resorufin by 0.5 U·mL^−1^ horseradish peroxidase (HRP) added to the medium. The activation of HRP is dependent on H_2_O_2_ produced by RLMt. Resorufin is a fluorescent derivative detected by a spectrofluorimeter operating at 563 nm for excitation and 587 nm for emission. This technique displays good signal/noise ratios and little artifactual interference 55, 56. Indeed, controls conducted in the absence of mitochondria or the absence of peroxidase indicate that nonspecific probe oxidation is negligible (<1% of the increment observed in the presence of mitochondria and peroxidase). Also, fluorescence increments are largely suppressed (>90%) in the presence of added catalase, indicating the response is mostly due to H_2_O_2_ production. Calibration was conducted by adding H_2_O_2_ at known concentrations (ε_240 nm_ = 43.6 m ^−1^·cm^−1^).

### Expression of recombinant wild‐type and mutant rat TOP

Clones construction, mutant generation, expression, and purification were performed as already published with some modifications 57. Briefly, recombinants wild‐type and mutant rTOP were expressed in Escherichia coli BL21(DE3) strain (Merck‐Novagen, Darmstadt, Germany) as a poly(His) tag fusion protein using the expression vector pET28a(+) (Merck‐Novagen). Protein purification was performed by affinity chromatography. The purity of the protein was determined by Coomassie brilliant blue staining on 12% SDS/PAGE. rTOP homogeneity was larger than 95% (data not shown). Protein samples were stored at −80 °C in small aliquots.

### Enzyme reduction

Preparations of purified recombinant rTOP were treated with the sulfhydryl reductant TCEP (1–10 mm) 58. After incubation, rTOP preparations were passed through a PD‐10 desalting column (GE Healthcare Life Sciences, Buckinghamshire, UK) to remove the reducing agents completely. After determination of protein concentration by absorbance (ε_280 nm_=78,115 m ^−1^·cm^−1^), aliquots of reduced rTOP preparations were taken for further incubation, hydrolytic assays, determination of TOP‐reduced Cys residues.

### Determination of rTOP‐reduced Cys residues

These assays were carried out as previously described 59. Briefly, 150–250 μg of rTOP was resuspended in 300 μL of 30 mm Tris (pH 7.4) containing 8 M urea. After complete dissolution, samples were taken for reading at 280 nm. Afterward, 10 μm DTNB (final concentration, Sigma‐Aldrich‐Merck) was added to the samples and incubated for 40 min in the dark followed by reading at 412 nm. Protein concentration was calculated as described below. The concentration of the CyS–TNB complex was calculated from the ε_412 nm_=14,150 m ^−1^·cm^−1^). The number of reduced Cys residues was calculated by the molar ratio protein/CyS–TNB complex.

### SDS/PAGE analysis

Electrophoresis was performed on 12% polyacrylamide gels containing sodium dodecyl sulfate 60. The samples were prepared under reducing and nonreducing conditions, depending on the presence of β‐mercaptoethanol in the sample buffer. Proteins were visualized with Coomassie Blue. All reactions were assayed at room temperature (25 °C) using Tris buffer pH 7.4 (Tris 50 mm, NaCl 100 mm). The reactions were stopped by ice‐cold freezing. 1–2 μm of thimet oligopeptidase were incubated with increasing concentrations of a reactant or fixed concentration. The SDS/PAGE gels were scanned, and the densitometry of protein bands on gel images was performed using the software imagej 61. The acquired data were further analyzed using grafit software (v.5.0, erithacus Software) 62.

### Western blot

For the western blot, 1 μg of recombinant TOP and 30 μg of cell extract proteins per lane were separated by 12% SDS/PAGE and electrotransferred to a PVDF membrane Hybond‐P (GE Healthcare) and then blotted with primary antibodies according to the manufacturer's instructions anti‐24.15 (anti‐THOP, Proteimax Biotechnology, São Paulo, Brazil) or anti‐GAPDH (Proteimax Biotechnology) detected by an anti‐rabbit secondary antibody (T2767, Invitrogen Corporation, Carlsbad, CA, USA). Data were analyzed by western blot densitometry using imagej software 61.

### Quantitative PCR

For the quantitative real time, RNA was isolated from cell plates using TRIzol Reagent (Thermo Fisher Scientific Inc., Waltham, MA, USA), according to the manufacturer's instructions. Two micrograms of total RNA were treated with DNase (Ambion Inc., Austin, TX, USA) and reverse‐transcribed into cDNA using Superscript III transcriptase and random primers (Invitrogen Corp.), according to the manufacturer's instructions. An aliquot of the cDNA (10 ng) was used as template for qPCR amplification. qPCRs were carried out in a 12‐μL PCR containing 3.2 pmol of each specific primer, 1x SYBR Green Master Mix (Thermo Fisher Scientific Inc.), and DEPC water. The PCR running parameter was the standard program in the ABI Prism 7500 sequence detection system (Thermo Fisher Scientific Inc.). The ΔΔCT was calculated relative to control samples and GAPDH reference gene using the comparative cycle threshold (Ct) method (2^ΔΔCt^), where ΔΔCt = ΔCt test sample − ΔCt control samples and ΔCt = Ct test sample − Ct reference gene) following the MIQE guidelines 63. The following qPCR primers were used: TOP Fw 5′‐CTCCCCAGAGAGACTCAG‐3′, TOP Rv 5′‐GTCGTGTCCTCGTTCAGGTT‐3′, GAPDH Fw 5′‐ACCACAGTCCATGCCATCAC‐3′ and GAPDH Rv 5′‐TCCACCACCCTGTTGCTGTA‐3′ (IDT, Integrated DNA Technologies).

### Enzyme activity assay

The hydrolysis of the fluorogenic substrates Abz‐GFSPFRQ‐Eddnp was performed as previously described 64, 65 in Tris 50 mm, NaCl 100 mm buffer, pH 7.4, at 37 °C. In the following, we measured the fluorescence at λ_em_ = 420 nm/λ_ex_ = 320 nm in a spectrofluorometer (F‐7000, Hitachi, Tokyo, Japan). The plate with 1 cm path length, containing 300 μL of the substrate solution was placed in a thermostatically controlled cell compartment for 5 min. In the following, the enzyme solution was added to the medium and the kinetic of fluorescence recorded for 5–10 min. The data were fitted, and the slope converted into Moles of hydrolyzed substrate per minute based on the fluorescence curves of standard peptide solutions before and after total enzymatic hydrolysis. The concentration of the peptide solutions was calculated from the colorimetric determination of the 2,4‐dinitrophenyl group (ε_365 nm_ = 17,300 m ^−1^·cm^−1^). The enzyme concentration for initial rate determination was chosen for hydrolyzing less than 5% of the substrate present. All the obtained data were fitted by nonlinear least squares equations, using grafit software (v.5.0, erithacus Software) 62.

### Trapping of sulfenic acid with NBD‐Cl

Protein (10 μm) was exposed to oxidants (90 μm) for 30 min. NBD‐Cl (Sigma‐Aldrich, 100 μm) was added to the samples and was incubated for 30 min, at 37 °C, after the samples were subjected to spin column (Amicon Ultra 10 kDa, Millipore, Ireland). Electronic absorption spectra were carried out in a photodiode Multispec 1501 spectrophotometer (Shimadzu Scientific Instruments, Columbia, MD), using quartz cuvettes with 1.0‐ and 0.1‐cm optical path and 0.5‐nm slit 66.

## Author contributions

MYI and JCF designed and performed the assays, analyzed the data, and wrote the manuscript. CHY, LVB, AM, and JMG performed the assays and analyzed the data. VO and ILN designed the work, analyzed the data, and wrote the manuscript.
